# Draft genome sequence of a docosahexaenoic-acid-producing marine protist, thraustochytrid strain 12B

**DOI:** 10.1128/MRA.00270-23

**Published:** 2023-08-14

**Authors:** Takehiko Sahara, Takumi Adachi, Tadashi Nagamine, Toru Fukano, Naoki Morita

**Affiliations:** 1 Bioproduction Research Institute, National Institute of Advanced Industrial Science and Technology (AIST), Tsukuba, Ibaraki, Japan; 2 Laboratory of Molecular Environmental Microbiology, Graduate School of Agriculture, Hokkaido University, Sapporo, Hokkaido, Japan; 3 Bioproduction Research Institute, National Institute of Advanced Industrial Science and Technology (AIST), Sapporo, Hokkaido, Japan; 4 ROM Co. Ltd, Sapporo, Hokkaido, Japan; Montana State University, Bozeman, Montana, USA

**Keywords:** thraustochytrid, draft genome, marine protist, eukaryote

## Abstract

We sequenced the draft genome of thraustochytrid strain 12B. This strain shows a high production of polyunsaturated fatty acids, such as docosahexaenoic acid. The draft genome sequence of approximately 65 Mbp will provide insights into metabolic engineering to improve the production of polyunsaturated fatty acids in the microorganism.

## ANNOUNCEMENT

A marine protist, thraustochytrid strain 12B (strain 12B) belonging to the family Thraustochytriaceae, was isolated from the mangrove area of Okinawa, Japan (1). Studies on the taxonomic characterization of strain 12B have not been completed; however, strain 12B has particularly interesting characteristics because of its ability to produce a significant amount of polyunsaturated fatty acids (PUFAs) (1, 2). Recently, an efficient transformation method has been developed (3), which could be one of the crucial techniques for the metabolic engineering of strain 12B. This will improve the production of PUFAs and other useful materials. Therefore, to better understand the unique characteristics of efficient PUFA production and to utilize genetic information to improve its productivity, we performed whole-genome sequencing of strain 12B.

Strain 12B was obtained from the Biological Resource Center, National Institute of Technology and Evaluation (NBRC), Japan, as strain number NITE P-68, and was cultivated in BY+790 medium consisting of 50% artificial seawater (My Sea, Jamarine Laboratory, Osaka, Japan), 1 g/L Bacto peptone (BD Bioscience, Flanklin Lakes, NJ), 1 g/L Bacto yeast extract (BD Bioscience), and 0.5 g/L d-glucose (FUJIFILM Wako Pure Chemical Corporation, Osaka, Japan) at 30°C for 2 days. Genomic DNA was prepared using the DNAs-ici!-F reagent (RIZO Inc., Ibaraki, Japan). The genomic DNA was quantified using the Qubit double-stranded DNA assay (Thermo Fisher Scientific, Waltham, MA). For whole-genome sequencing, a 400-bp fragment library was prepared from 1 µg of the genomic DNA using the Ion Xpress Plus Fragment Library Kit (Thermo Fisher Scientific). The library was used to prepare a templated-beads sample by amplification and enrichment using the Ion PGM Hi-Q OT2 Kit (Thermo Fisher Scientific) on the Ion OneTouch 2 system (Thermo Fisher Scientific). The templated-beads sample was manually loaded onto the Ion 318 Chip v2 BC (Thermo Fisher Scientific) and sequenced on the Ion PGM system (Thermo Fisher Scientific) with the Torrent Suite software (ver. 5.0.3) by using the Ion PGM Hi-Q Sequencing Kit (Thermo Fisher Scientific).

As a result of the sequencing, 4,364,157 trimmed and filtered reads (mean length: 288 bp, total length: 1.25 Gbp, and mean quality value: 27.6) were obtained using the Torrent Suite software. The reads were used for *de novo* assembly using the MIRA software ver. 4.0.5 (4), and 4,694 contigs (average coverage of 20.2×) were assembled. The total contig length was 65,901,411 bp, the largest contig length was 158,719 bp, and the N_50_ value was 27,391 bp. The overall GC content value was 45.1%. Using the BUSCO software ver. 5.5.4 (5) with the eukaryote_odb10 data set (2020-09-10), genome completeness was estimated to be 86.3% (82.0% single copy and 4.3% duplicated). Gene prediction was performed using the AUGUSTUS software ver. 3.3.1 (6). The 13,365 predicted proteins were searched against the nr protein database (v4, 2020-02-03) of the National Center for Biotechnology Information using the Blast2GO software ver. 6.0.3 (7), and matches were found for 11,698 proteins at an E-value cutoff of 10^−4^. Default parameters were used on analysis software except where otherwise noted.

## Data Availability

The nucleotide sequences of the thraustochytrid strain 12B draft genome were deposited in DDBJ/ENA/GenBank under the accession numbers BSEB01000001 to BSEB01004694. The version described in this paper is the first version. The sequencing reads have been deposited in the DDBJ Sequence Read Archive under the accession number DRR426623. The BioProject and BioSample accession numbers are PRJDB14942 and SAMD00567560, respectively.
